# Guided migration analyses at the single-clone level uncover cellular targets of interest in tumor-associated myeloid-derived suppressor cell populations

**DOI:** 10.1038/s41598-020-57941-8

**Published:** 2020-01-27

**Authors:** Silvia Duarte-Sanmiguel, Vasudha Shukla, Brooke Benner, Jordan Moore, Luke Lemmerman, William Lawrence, Ana Panic, Shipeng Wang, Nicholas Idzkowski, Gina Guio-Vega, Natalia Higuita-Castro, Samir Ghadiali, William E. Carson, Daniel Gallego-Perez

**Affiliations:** 1 Department of Biomedical Engineering, The Ohio State University, Columbus, OH USA; 2 OSU Nutrition, The Ohio State University, Columbus, OH USA; 3 Biomedical Sciences Graduate Program, The Ohio State University, Columbus, OH USA; 4 Department of Surgery, The Ohio State University, Columbus, OH USA; 5 Department of Medicine, National University of Colombia, Bogota, Colombia

**Keywords:** Surgical oncology, Cancer microenvironment

## Abstract

Myeloid-derived suppressor cells (MDSCs) are immune cells that exert immunosuppression within the tumor, protecting cancer cells from the host’s immune system and/or exogenous immunotherapies. While current research has been mostly focused in countering MDSC-driven immunosuppression, little is known about the mechanisms by which MDSCs disseminate/infiltrate cancerous tissue. This study looks into the use of microtextured surfaces, coupled with *in vitro* and *in vivo* cellular and molecular analysis tools, to videoscopically evaluate the dissemination patterns of MDSCs under structurally guided migration, at the single-cell level. MDSCs exhibited topographically driven migration, showing significant intra- and inter-population differences in motility, with velocities reaching ~40 μm h^−1^. Downstream analyses coupled with single-cell migration uncovered the presence of specific MDSC subpopulations with different degrees of tumor-infiltrating and anti-inflammatory capabilities. Granulocytic MDSCs showed a ~≥3-fold increase in maximum dissemination velocities and traveled distances, and a ~10-fold difference in the expression of pro- and anti-inflammatory markers. Prolonged culture also revealed that purified subpopulations of MDSCs exhibit remarkable plasticity, with homogeneous/sorted subpopulations giving rise to heterogenous cultures that represented the entire hierarchy of MDSC phenotypes within 7 days. These studies point towards the granulocytic subtype as a potential cellular target of interest given their superior dissemination ability and enhanced anti-inflammatory activity.

## Introduction

The tumor microenvironment is highly heterogeneous in nature, with cancerous cells co-habiting with both stromal and immune cells. Such complex cellular interplay plays a central role in modulating tumor progression. Myeloid-derived suppressor cells (MDSCs), in particular, have been known to exert immunosuppressive activity in the tumor niche, which protects cancerous cells from the host immune system and/or different therapeutic modalities^1,2^. While a lot of research has been devoted to developing advanced drugs and drug delivery systems to target cancerous cells^3–5^, and/or blocking MDSC-driven immunosuppression within the tumor niche^6,7^, less is known about the motility mechanisms by which MDSCs disseminate and colonize the tumor in the first place.

MDSCs are innate immune cells that are highly expanded in cancer patients^2^. These cells tend to infiltrate tumors and lymphoid tissues, and their levels correlate with increased tumor burden and limited survival in a variety of malignancies^6–9^. MDSCs specifically contribute to the loss of immune effector cell function and reduce the efficacy of immunotherapies. As such, MDSCs have emerged as an attractive therapeutic target in cancer. Drugs that inhibit MDSC effector functions or proliferation within the tumor could potentially lead to an enhanced host anti-tumor immune response and clearance of the cancer burden. However, efforts to effectively target MDSCs within the tumor niche have been hampered by a lack of robust “druggable” targets at the cellular and/or molecular level. While targeting the dissemination-based mechanisms by which MDSCs infiltrate the tumor niche could be a viable alternative strategy against MDSC-driven immunosuppression at the tumor site, our understanding of such mechanisms for MDSCs is limited compared to what we know about the dissemination modalities of cancerous tumor cells. Structurally guided migration has been known to play a key role in the escape of cancerous cells from the primary tumor, as well as in dissemination and metastasis^10–15^. Nevertheless, to the best of our knowledge, no study has probed MDSC motility under structurally guided dissemination conditions. Here we used microscale engineering tools, coupled with cellular and molecular biology analysis tools, to probe the dissemination capabilities of MDSCs at the single-clone level under guided migration conditions, and to identify MDSC subpopulations of interest based on their disseminative and suppressive capabilities.

## Results and Discussion

### MDSCs respond to topographical cues and exhibit structurally guided dissemination patterns

**Figure 1:**
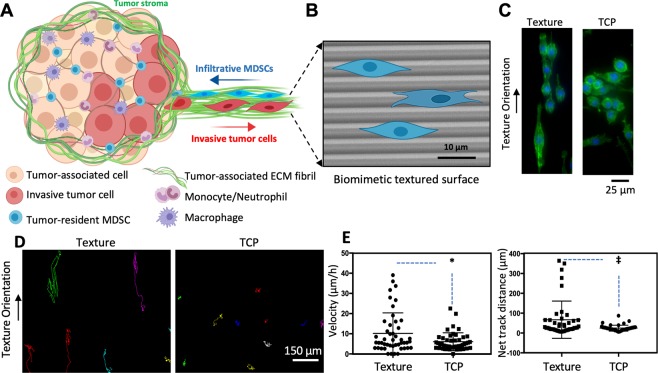
MDSCs are responsive to aligned structural cues and exhibit guided dissemination patterns. (**A**) Schematic diagram of the tumor microenvironment showing invasive cancer cells and infiltrative MDSCs using pre-aligned structural cues (*e.g*., remodeled ECM, blood vessel walls) to escape and invade the tumor stroma, respectively. (**B**) SEM micrograph (with superimposed MDSC mock-ups) of a PDMS-based biomimetic textured surface used to evaluate structurally guided MDSC migration at the single-clone level. (**C**) Actin (green) – Nuclei (blue) staining of MDSCs cultured on textured vs. control/TCP surfaces. MDSCs assume an aligned/more migratory morphology on the textured surfaces compared to TCP. (**D**) Single-clone dissemination tracks and (**E**) quantification of MDSCs on textured vs. control/TCP surfaces confirming enhanced dissemination capabilities (*i.e*., average single-clone velocity and net track distance) for MDSCs when exposed to pre-aligned structural cues. The net track distance is a reflection of the geometrical distance traveled by a cell during the tracking period. *p < *0.01* and ^‡^*p* < *0.02* (t-test, n = 4).

Structurally guided cell dissemination has been known to play a role in the escape of cancerous cells from the primary tumor and the establishment of metastatic outgrowths in peripheral organs and tissues. Highly aggressive cancer cells tend to exhibit distinct spreading patterns, disseminating preferentially along pre-aligned anatomical microstructures within the tissues, including radially oriented fibrils from the extracellular matrix (ECM), white matter tracts, the basal lamina of blood vessels, and the subpial/subperitoneal spaces, among others (Fig. 1A)^11,16,17^. Micro- and nanoscale tools have been used to develop systems that can be utilized to probe cancer cell motility under these physiologically relevant conditions^11,17–20^. While topographical or cell confinement cues have been used to mimic rapid and highly directional motility in a wide variety of cancerous cells^11,17,18,21–23^, to the best of our knowledge, no studies have looked into the influence of such cues on the dissemination/infiltration capabilities of tumor-associated MDSCs. Here we tested whether MDSCs respond to topographical cues by exhibiting structurally guided dissemination patterns similar to invasive cancerous cells. The murine MDSC cell line, MSC-2, was used as a model^6,24^. These cells were plated on microtextured polydimethylsiloxane (PDMS) surfaces (Fig. 1B), which were fabricated via replica molding from photolithographically fabricated silicon masters, and were designed as an array of parallel ridges and grooves with dimensions that have been previously tested in cancer cell dissemination studies (~2 µm × 2 µm with 2 µm spacing)^10–13^. MDSC motility was monitored at the single-clone level in real time via time-lapse microscopy. Cells plated on a standard cell culture surface (*i.e*., tissue culture polystyrene or TCP) were used for comparison purposes. Our results indicate that MDSCs show limited motility at the single-clone level on TCP (Fig. 1C–E) (Video S1), with most cells exhibiting a rounded morphology (Fig. 1C). Textured surfaces, on the other hand, clearly induced cytoskeletal and morphological rearrangements (*i.e*., alignment) in some of the MDSCs (Fig. 1C), which were conducive to increased motility (Fig. 1D,E) (Video S2). Average single-clone velocities reached a maximum of ~40 μm h^−1^ on textured surfaces compared to ~20 μm h^−1^ on TCP. Net track distances, which are a measure of the effective displacement of a single clone, reached a maximum of ~400 μm over a period of 16 hours on textured surfaces compared to <100 μm on TCP. Notably, MDSCs migrating on textured surfaces exhibited significant inter-clonal variability in the dissemination potential, with cells spanning the whole spectrum from low to high motility. In contrast, MDSCs migrating on TCP showed markedly less inter-clonal variability. Studies with circulating MDSCs derived from cancer patients (Fig. S1) further confirmed the existence of highly motile MDSC populations exhibiting marked inter-clonal variability, with some clones showing average guided migration velocities of up to ~200 μm h^−1^, and total net displacements that approached 1 mm over a period of 16 hours. However, we also found that certain populations of patient-derived circulating MDSCs exhibited limited overall motility, which could potentially be a direct reflection of the underlying malignancy (*e.g*., type, stage, mutations) and/or concurrent treatment modalities (Tables S1–S3).

### MDSC subpopulations exhibit different dissemination capabilities

**Figure 2:**
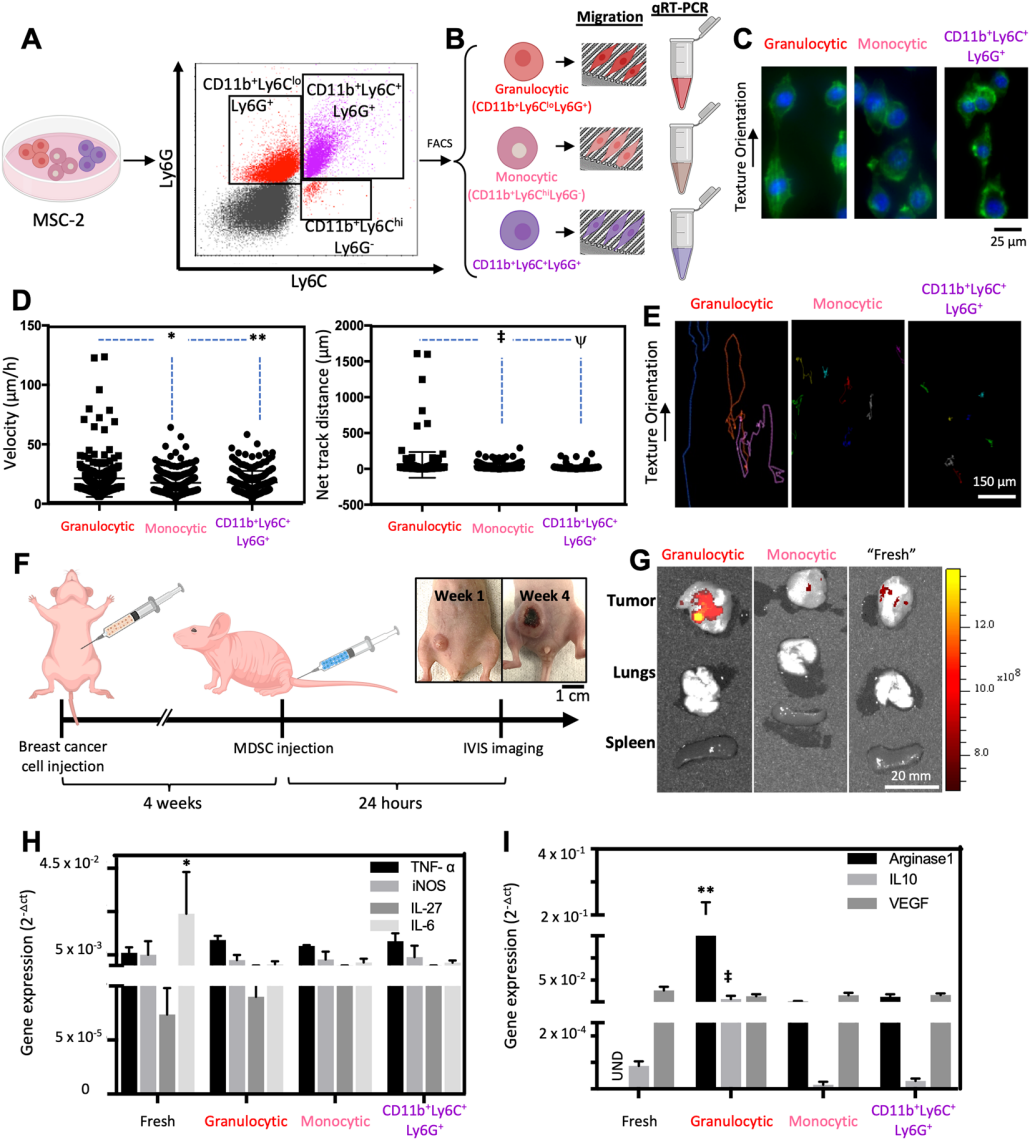
MDSCs subpopulations exhibit distinct dissemination and gene expression patterns. (**A,B**) Schematic diagram of the experimental design. Here MSC-2 cultures were sorted by flow cytometry into three distinct subpopulations, including granulocytic (CD11b^+^Ly6C^lo^Ly6G^+^) and monocytic (CD11b^+^Ly6C^hi^Ly6G^−^) MDSCs, as well as CD11b^+^Ly6C^+^Ly6G^+^ cells. Each population was then subjected to single-clone motility assays on textured PDMS and qRT-PCR analyses of pro- and anti-inflammatory markers. (**C**) Actin (green) – Nuclei (blue) staining of different MSC-2 subtypes cultured on textured surfaces. Granulocytic MDSCs had a tendency to exhibit a more aligned and migration-prone morphology compared to their counterparts. (**D**) Single-clone dissemination (*i.e*., average velocities and net track distances) quantification for each subtype on textured surfaces. *p = *0.006*, **p < *0.001*, ^ψ^*p* *=* *0.001*, ^‡^*p* *=* *0.09* (2-way ANOVA, n = 4). (**E**) Single-clone tracks for each population. (**F**) Fluorescently labeled flow-sorted MDSCs vs. “fresh”/unsorted MDSCs were injected (*i.e*., via the tail vein) into tumor-bearing mice (*i.e*., orthotopic breast tumor developed from human cells in nude mice). Photographs to the right depict tumor progression/growth from week 1 to week 4. (**G**) The mice were sacrificed 24 hours post-injection, and the tumors and other target organs were imaged to detect the degree of MDSC infiltration. qRT-PCR analysis of (H) pro-inflammatory and (I) anti-inflammatory genes for each subtype. *p < *0.001*, **p < *0.0001*, ^‡^*p* = *0.03* (2-way ANOVA, n = 3–4).

Based on the clear inter-clonal variability in motility, we proceeded to further stratify and probe the MDSC population via flow cytometry-based sorting into granulocytic (CD11b^+^Ly6C^lo^Ly6G^+^) and monocytic (CD11b^+^Ly6C^hi^Ly6G^−^) subpopulations (Fig. 2A–C) based on standard MDSC nomenclature^25^. A subpopulation of CD11b^+^Ly6C^+^Ly6G^+^ cells was also identified from the flow cytometry data and included in our analyses. Flow-sorted subpopulations were then subjected to structurally guided motility studies on textured surfaces, as described above, in addition to qRT-PCR analyses of pro- and anti-inflammatory markers. Single-clone dissemination studies indicate that when probed in isolation, granulocytic MDSCs have superior dissemination capabilities compared to monocytic MDSCs and the CD11b^+^Ly6C^+^Ly6G^+^ subpopulation (Fig. 2D) (Videos S3–5), with single clones reaching in some cases average velocities and net displacements of >100 μm h^−1^ and ~1.5 mm over a period of 16 hours. And while some clones within the monocytic MDSC and CD11b^+^Ly6C^+^Ly6G^+^ subpopulations showed relatively high average migration velocities, ~50 μm h^−1^, net displacements were considerably limited, thus suggesting that these cells tend to show very short range and/or disorganized motility patterns compared to granulocytic MDSCs (Fig. 2E). These observations were further confirmed via *in vivo* studies (Fig. 2F,G), where tumor-bearing mice were systemically injected with fluorescently labeled suspensions of sorted vs. “fresh”/unsorted MDSCs, and IVIS was used to document MDSC accumulation within the tumor niche vs. peripheral organs/tissues. The mice that were injected with granulocytic MDSCs showed more pronounced fluorescence signal accumulation within the tumor (Fig. 2G). Parallel single-clone motility studies with circulating MDSCs derived from cancer patients (Fig. S2) also suggest that the granulocytic subpopulation (CD11b^+^CD15^+^CD14^−^) exhibits enhanced motility compared to the monocytic one (CD11b^+^CD15^−^CD14^+^). Gene expression analysis of pro-inflammatory markers indicate no statistically significant differences in the expression of *TNF-α*, *iNOS*, and *IL-27* between the “fresh” (*i.e*., unsorted) MDSC population and the purified granulocytic, monocytic, and CD11b^+^Ly6C^+^Ly6G^+^ subpopulations. However, *IL-6* was significantly overexpressed in the fresh population vs. the flow-sorted subpopulations. Gene expression analysis of anti-inflammatory markers, on the other hand, suggest that the flow-sorted granulocytic subpopulation has a tendency to overexpress *arginase* and *IL-10* compared to the fresh and flow-sorted monocytic and CD11b^+^Ly6C^+^Ly6G^+^ subpopulations. Altogether, these results suggest that the granulocytic MDSC subpopulation appears to be not only more prone to disseminating and colonizing cancerous tissue, but also to overexpress anti-inflammatory/suppressive markers compared to the monocytic MDSC and the CD11b^+^Ly6C^+^Ly6G^+^ subpopulations.

### MDSC subpopulations show phenotypic plasticity that drives populational homeostasis under prolonged culture conditions

**Figure 3:**
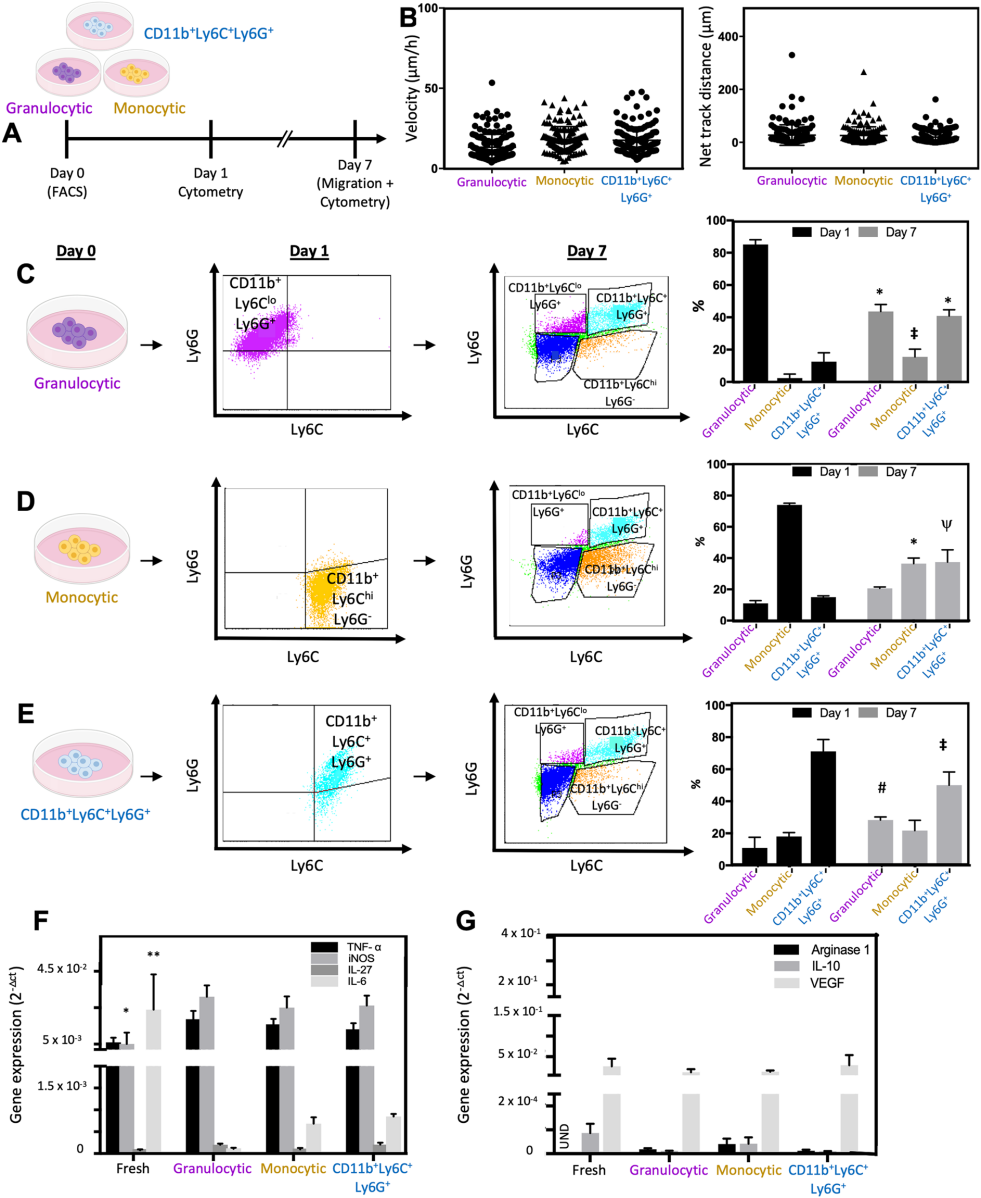
Single MDSC subpopulations appear to show phenotypic plasticity that can drive the replenishment the entire phenotypic spectrum. (**A**) Schematic diagram of the experimental design. (**B**) Single-clone dissemination (*i.e*., average velocities and net track distances) studies did not show significant differences between all three populations by day 7. (**C–E**) Flow cytometry analyses indicate that while by day 1 post-sorting all subpopulations remained relatively pure, by day 7 the entire spectrum of phenotypes had been replenished regardless of the phenotype of the starting cell population. *p < *0.0001*, ^‡^*p* = *0.01*, ^#^*p* = *0.03*, ^ψ^*p* = *0.0001* (2-way ANOVA/Tukey’s multiple comparisons, n = 3–4). qRT-PCR analyses of (F) pro-inflammatory and (**G**) anti-inflammatory genes at day 7 post-sorting. *p = *0.006*, **p = *0.01* (2-way ANOVA/Tukey’s multiple comparisons, n = 3–6).

Following flow-based purification of the MSC-2 cells into distinct subpopulations of granulocytic and monocytic MDSCs, as well as CD11b^+^Ly6C^+^Ly6G^+^ cells, the cells were maintained in culture for 1–7 days. Phenotypic plasticity was evaluated via flow cytometry at days 1 and 7. Single-clone motility assays and gene expression analyses were run at day 7 (Fig. 3A). Surprisingly, and in contrast to what we found immediately after flow-based sorting; no significant differences were detected in the dissemination characteristics across all three populations by day 7 (Fig. 3B). Average single-clone velocities stayed within ~50 μm h^−1^ for all populations, while the overall net track distance stayed below ~200 μm. Flow cytometry analyses indicated that 1 day post-sorting the purified populations still comprised the majority (~80%) of the culture, however, by day 7 the whole hierarchy of populations had been reestablished (Fig. 3C–E), possibly suggesting a role for cellular plasticity in the maintenance of populational homeostasis/heterogeneity in MDSC populations. Cell cultures derived from the purified granulocytic subpopulation (Fig. 3C), for example, gave rise to monocytic MDSCs and CD11b^+^Ly6C^+^Ly6G^+^ cells, with the monocytic subpopulation showing the sharpest increase from day 1 to 7 (~7-fold change), and the CD11b^+^Ly6C^+^Ly6G^+^ population showing a ~3-fold increase by day 7. Cultures derived from purified monocytic MDSCs, on the other hand, were more prone to giving rise to the CD11b^+^Ly6C^+^Ly6G^+^ population by day 7 (~2.5-fold increase) compared to the granulocytic population. Finally, cultures derived from the purified CD11b^+^Ly6C^+^Ly6G^+^ population were more prone to giving rise to granulocytic MDSCs by day 7 (~3-fold increase) compared to the monocytic MDSCs, which did not show a significant increase between days 1 and 7. Gene expression profiles of pro- (Fig. 3F) and anti-inflammatory (Fig. 3G) markers at day 7 showed more subtle differences across populations, with decreased and increased *iNOS* and *IL-6* expression, respectively, in the “fresh” MDSC population relative to the sorted/purified subpopulations. However, when comparing the expression profiles between day 0 (*i.e*., day of sorting/purification) and day 7, a more pronounced difference was noted, with an overall increase in the expression of pro-inflammatory *iNOS* for all three populations, and a significant decrease in *arginase1* and *Il-10* for the granulocytic subpopulation only (Fig. S3).

## Conclusions

Micro- and nanoscale technologies have been used extensively to probe and/or modulate various aspects of cell biology for medical applications^10–15,26–36^, especially in cancer therapy and diagnostics^3,37–42^. Here we used microscale engineering tools to demonstrate that tumor-associated MDSCs exhibit structurally guided migration patterns, similar to invasive cancerous cells. Single-clone motility analyses unmasked clear heterogeneities within and across (*i.e*., for patient-derived MDSCs) MDSC populations, confirming the presence of clonal subsets with enhanced dissemination capabilities in both murine and patient-derived MDSCs. Follow-up motility studies coupled with flow cytometry-based sorting, gene expression analyses, and orthotopic tumor xenograft experiments in nude mice, suggest that the granulocytic subpopulation is more prone to exhibiting increased dissemination and tumor-infiltrative ability, as well as enhanced anti-inflammatory activity, which could make this population an attractive cellular target in cancer research and therapeutic development. Subsequent studies, however, highlight the remarkably dynamic and plastic nature of such clonal subsets, with purified MDSC subpopulations quickly reaching populational homeostasis by giving rise to the full spectrum of MDSC phenotypes. While there have been conflicting reports regarding the dominant phenotype of tumor-resident MDSCs (*i.e*., granulocytic vs. monocytic)^43–47^, our single-clone dissemination and phenotypic plasticity results point towards a potential mechanism by which granulocytic MDSCs are presumably better equipped to infiltrate the tumor niche, where they could then remain as granulocytic and/or give raise to monocytic MDSCs depending on multiple factors, including the tumor type. Interestingly, single-clone dissemination studies with circulating MDSCs derived from cancer patients suggest that MDSC motility could potentially be impacted by the patient’s background (*e.g*., type/stage of cancer, treatment modalities, etc.), and as such, additional studies are needed to determine whether the dissemination patterns of circulating MDSCs, *ex vivo*, could be used to monitor disease and/or treatment progression.

## Materials and Methods

### Textured PDMS surfaces

Microtextured PDMS surfaces were fabricated from photolithographically patterned silicon masters via a replica molding process. A parallel array of ridges and grooves (2 µm wide, 2 µm tall, spaced by 2 µm) was first patterned on a silicon master via standard UV photolithography using S1813 photoresist. A 10:1 mixture of PDMS with curing agent was then cast on the master and allowed de-gas and cure for several hours. The PDMS was then demolded from the master, sterilized and placed on multi-well plates for single-cell migration experiments. Scanning electron microscopy (SEM) was used to characterize the surface morphology.

### MDSC cultures

The mouse MDSC cell line (MSC-2) was a kind donation from Gregoire Mignot. MSC-2 cells were cultured in RPMI 1640 media supplemented with 25 mM HEPES, 10% heat-inactivated fetal bovine serum (FBS), 1% antibiotic-antimycotic, and 1 mM sodium pyruvate. Patient-derived MDSCs were enriched from peripheral blood using the RosetteSep HLA-myeloid cell enrichment kit (Stemcell Technologies) followed by Ficoll-Paque centrifugation (GE healthcare). MDSC were isolated by subsequent negative selection of HLA-DRneg cells using anti-HLA-DR MicroBeads (Miltenyi Biotec) for 15 minutes at 4 °C and isolated using a MS-MACS column. Patient-derived MDSCs were acquired with informed consent under institutional review board (IRB)-approved protocols for human subject research at The Ohio State University, in accordance with the Declaration of Helsinki.

### Single-cell migration assays

~1.5 × 10^5^ MSC-2 cells were seeded and allowed to adhere on the textured PDMS surfaces or TCP controls in regular culture media for several hours. Cells were imaged via time-lapse microscopy every 10 minutes for over 16 h using a cell culture chamber (Okolab) mounted on an inverted microscope. Images were analyzed using the manual tracker plugin in Fiji. Single-cell displacement data were then analyzed via MATLAB to determine velocities and net track traveled distances.

### Flow cytometry-based analysis and sorting

The following antibodies were used for the MSC-2 cells: anti-CD11b-FITC, anti-Ly6-C-APC and anti-Ly6-G-PE, all purchased from Biolegend. For patient-derived MDSCs, we used anti-CD33-APC, anti-CD11b-AP, and anti-HLA-DR-PECy7, purchased from Beckman Coulter. Data were acquired using an LSRII flow cytometer (BD Biosciences). All colors were evaluated against their respective isotype controls and samples with no staining.

### Gene expression analyses

Total RNA was extracted using the TRizol reagent (ThermoFisher). Reverse transcription reactions were performed using 500–1000 ng RNA in a 20 μl reaction with the superscript VILO cDNA synthesis kit (ThermoFisher). cDNA was used as a template to measure the expression levels of pro- and anti-inflammatory genes by quantitative real-time PCR using predesigned primers. Real-time PCR reactions were performed using the QuantStudio 3 Real-Time PCR System with TaqMan fast advance chemistry (Thermo Scientific) with the following conditions: 95 °C 10 min, 40 cycles of 95 °C 1 min, 60 °C 1 min, and 72 °C 1 min. Gene expression was normalized against the house keeping genes GAPDH and ATP-6.

### Orthotopic tumor xenografts

Immunodeficient nude mice (Jackson Laboratory), 6–8-week-old, were first injected with 1 million human breast cancer cells (MDA-MB-231, ATCC) in the mammary fat pad to generate tumors. After 4 weeks of tumor development, sorted MDSC subpopulations were stained using PKH67 green fluorescent cell linker kit for general cell membrane labeling (Millipore Sigma) following the instructions suggested by the manufacturer. Tumor-bearing mice were then injected with ~2.5 × 10^5^ MDSCs via the tail vein. The mice were then collected 1-day post-injection, and the tumors, lungs and spleens were characterized with an IVIS Imaging System (Xenogen Imaging Technologies). All animal studies were performed in accordance with protocols approved by the Laboratory Animal Care and Use Committee of The Ohio State University.

### Statistical analysis

All statistical analyses were run in Sigma Plot 12 or GraphPad Prism 7. We used n = 3–6 replicates per experiment. Specific information on the number replicates, statistical tests, and levels of significance can be found in the figure legends.

## Supplementary information

Supplementary Information.

Supplementary Video 1.

Supplementary Video 2.

Supplementary Video 3.

Supplementary Video 4.

Supplementary Video 5.

## Data Availability

The data generated through this study are available from the corresponding author upon reasonable request.
