# Supplementary material for: Guided migration analyses at the single-clone level uncover cellular targets of interest in tumor-associated myeloid-derived suppressor cell populations
Source: Sci Rep. 2020 Jan 27;10:1189. doi: 10.1038/s41598-020-57941-8 (PMC6985212; doi:10.1038/s41598-020-57941-8)
Supplement: Supplementary file 1 — Supplementary Information. [file 41598_2020_57941_MOESM1_ESM.pdf]

Supplementary Information

Guided migration analyses at the single-clone level uncover cellular targets of interest in tumor-associated myeloid-derived suppressor cell populations

*Silvia Duarte-Sanmiguel, Vasudha Shukla, Brooke Benner, Jordan Moore, Luke Lemmerman, William Lawrence, Ana Panic, Nicholas Idzkowski, Gina Guio-Vega, Natalia Higuita-Castro, Samir Ghadiali, William E. Carson, Daniel Gallego-Perez**

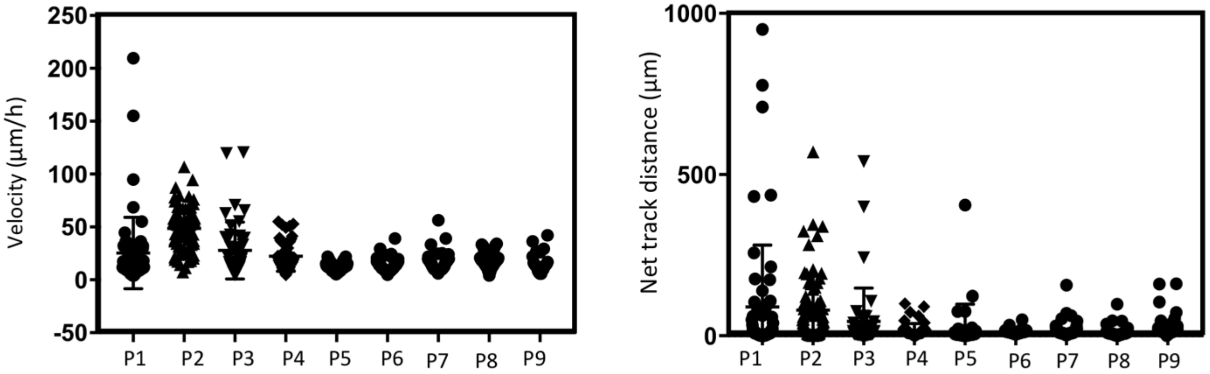

Figure S1. Circulating MDSCs derived from melanoma patients show different dissemination profiles at the single-clone level. Average single clone velocities (left) and net track distances (right) had a tendency to be significantly higher for certain patients compared to the rest of the patient population, which could be a reflection of the patient's background (Tables S1-S3).

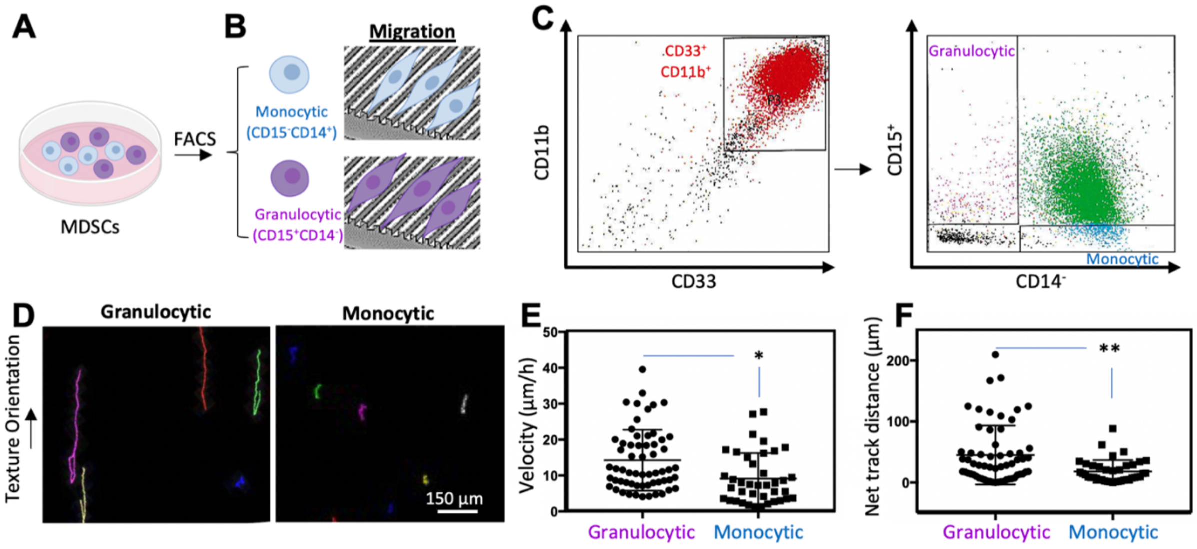

Figure S2. Distinct subpopulations of patient-derived MDSCs show different dissemination capabilities. (A-C) Melanoma patient MDSCs were sorted into granulocytic ($CD11b^{+}CD15^{+}CD14^{-}$) and monocytic ($CD11b^{+}CD15^{-}CD14^{+}$) subpopulations via flow cytometry. Similar to our observations in mouse MDSCs, the granulocytic subpopulation of patient-derived MDSCs also shows (D-F) increased dissemination (*i.e.,* average single-clone velocities and net track distances) capabilities compared to the monocytic subtype. *$p=0.0005$, **$p=0.002$ (Mann-Whitney, n= 3).

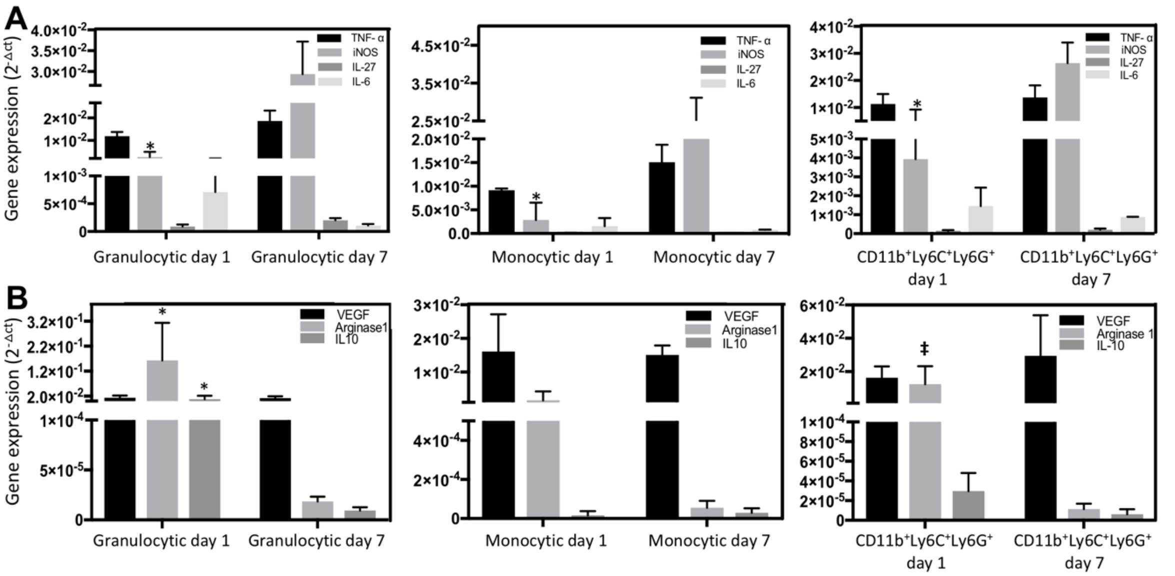

Figure S3. Differences in gene expression as a function of time for flow cytometry-sorted subpopulations. (A) Pro-inflammatory markers. (B) Anti-inflammatory markers. *$p<0.0001$, ‡$p=0.06$ (2-way ANOVA/Sidak's multiple comparisons, n= 3-6).

Table S1. Background information for MDSC samples obtained from cancer patients.

Patient ID	Malignancy	Stage	Mutation	Therapy
**P1**	Melanoma	IIIC	+BRAF V600	Nivolumab<br>Surgery
**P2**	Melanoma	IV	-BRAF	Nivolumab
**P3**	Melanoma	IV	+BRAF V600	Radiation<br>Pembrolizumab
**P4**	Melanoma	IV	-BRAF	Nivolumab<br>Ipilimumab
**P5**	Melanoma	IV	BRAF unknown	Pembrolizumab<br>Ipilimumab<br>Nivolumab
**P6**	Melanoma	IV	+BRAF V600	IFN-Alpha
**P7**	Melanoma	IV	NA	Leukine<br>Nivolumab
**P8**	Melanoma	IIB	NA	Nivolumab
**P9**	Melanoma	IIB	NA	Nivolumab

Table S2. Single-clone velocity comparisons across patients (One-way ANOVA/Tukey).

**Patient comparison**	**Significantly different?**	***p* value**
P1 vs. P2	Yes	<0.0001
P1 vs. P3	No	0.9993
P1 vs. P4	No	0.9967
P1 vs. P5	No	0.0582
P1 vs. P6	Yes	0.0423
P1 vs. P7	No	0.2149
P1 vs. P8	No	0.6429
P1 vs. P9	No	0.7526
P2 vs. P3	Yes	<0.0001
P2 vs. P4	Yes	<0.0001
P2 vs. P5	Yes	<0.0001
P2 vs. P6	Yes	<0.0001
P2 vs. P7	Yes	<0.0001
P2 vs. P8	Yes	<0.0001
P2 vs. P9	Yes	<0.0001
P3 vs. P4	No	0.9104
P3 vs. P5	Yes	0.0181
P3 vs. P6	Yes	0.0121
P3 vs. P7	No	0.0748
P3 vs. P8	No	0.3195
P3 vs. P9	No	0.4792
P4 vs. P5	No	0.3736
P4 vs. P6	No	0.3870
P4 vs. P7	No	0.7764
P4 vs. P8	No	0.9859
P4 vs. P9	No	0.9795
P5 vs. P6	No	>0.9999
P5 vs. P7	No	0.9976
P5 vs. P8	No	0.9122
P5 vs. P9	No	0.9972
P6 vs. P7	No	0.9998
P6 vs. P8	No	0.9516
P6 vs. P9	No	0.9996
P7 vs. P8	No	0.9992
P7 vs. P9	No	>0.9999
P8 vs. P9	No	>0.9999

Table S3. Single-clone net track distance comparisons across patients (One-way ANOVA/Tukey).

**Patient comparison**	**Significantly different?**	***p* value**
P1 vs. P2	No	0.9996
P1 vs. P3	No	0.2711
P1 vs. P4	Yes	0.0017
P1 vs. P5	Yes	0.0474
P1 vs. P6	Yes	0.0005
P1 vs. P7	Yes	0.0026
P1 vs. P8	Yes	0.0005
P1 vs. P9	No	0.5859
P2 vs. P3	No	0.5493
P2 vs. P4	Yes	0.0071
P2 vs. P5	No	0.1324
P2 vs. P6	Yes	0.0023
P2 vs. P7	Yes	0.0104
P2 vs. P8	Yes	0.0022
P2 vs. P9	No	0.8158
P3 vs. P4	No	0.8720
P3 vs. P5	No	0.9957
P3 vs. P6	No	0.7667
P3 vs. P7	No	0.9140
P3 vs. P8	No	0.7310
P3 vs. P9	No	>0.9999
P4 vs. P5	No	0.9999
P4 vs. P6	No	>0.9999
P4 vs. P7	No	>0.9999
P4 vs. P8	No	>0.9999
P4 vs. P9	No	0.97777
P5 vs. P6	No	0.9991
P5 vs. P7	No	>0.9999
P5 vs. P8	No	0.9981
P5 vs. P9	No	0.9995
P6 vs. P7	No	>0.9999
P6 vs. P8	No	>0.9999
P6 vs. P9	No	0.9518
P7 vs. P8	No	>0.9999
P7 vs. P9	No	0.9870
P8 vs. P9	No	0.9379
